# Water droplet-driven and perforated conducting polymer composite energy harvester: platform for powering portable and wearable electronics

**DOI:** 10.1039/d5ra04176g

**Published:** 2025-12-15

**Authors:** Vinod V. T. Padil, Sung-Ho Shin, Min Wook Pin, Joon Ha Chang, Hyeon Do Shin, Daniel M. Mulvihill, Jiamu Qian, Paolo Matteini, Byungil Hwang, Tae Gwang Yun, Jun Young Cheong, Il-Doo Kim

**Affiliations:** a School of Nanoscience (SNS), Central University of Gurajat (CUG) Kundhela-391107 Vadodara Gujarat India; b Department of Materials Science and Engineering, KAIST Daejeon 34141 Republic of Korea idkim@kaist.ac.kr; c Analysis and Assessment Research Group, Research Institute of Industrial Science and Technology (RIST) 37673 Republic of Korea; d Department of Materials Science and Engineering, Korea National University of Transportation Chungju 27469 Republic of Korea; e Department of Material Science and Engineering, Polymer Division, Graduate School of Chungnam National University 99 Daehak-ro, Yuseong District Daejeon Republic of Korea; f James Watt School of Engineering, University of Glasgow Glasgow G12 8QQ UK JunYoung.Cheong@glasgow.ac.uk; g Institute of Applied Physics “Nello Carrara”, National Research Council 50019 Sesto Fiorentino Italy; h School of Integrative Engineering, Chung-Ang University Seoul 06974 Republic of Korea bihwang@cau.ac.kr; i Department of Molecular Science and Technology, Ajou University Suwon 16499 Republic of Korea ytk0402@ajou.ac.kr

## Abstract

A major challenge for portable and wearable systems is reliable delivery of electrical power without use of external power supply. Herein, we exploit water droplets to produce electricity in the form of portable or wearable devices to operate electronic systems. An active polymeric layer composed of PVA [poly(vinyl alcohol)] with PSSA [poly(4-styrenesulfonic acid)] (a proton source) and PSSA-MA [poly(styrene sulfonic acid-*co*-maleic acid)] (cross-linking agent) is employed to charge the supercapacitor. By water absorption and ion diffusion, the energy conversion device reliably generates a DC output for long-term period. The Output performance of combined devices were simply increased up to 1.8 V in serial connection and 1.2 mA in parallel connection. Finally, the device is capable of storing electrical energy using supercapacitors of 220 mF up to 3.2 V, delivering power to a 60 mW practical device. This highlights the potential of a water-induced innovative power supply technology that is able to be integrated with the human body in portable and wearable forms for driving sensors and small electronics.

## Abstract 2

Water droplet-driven and perforated conducting polymer composite energy harvester is developed for sustainable energy generation and storage.

## Graphical abstract

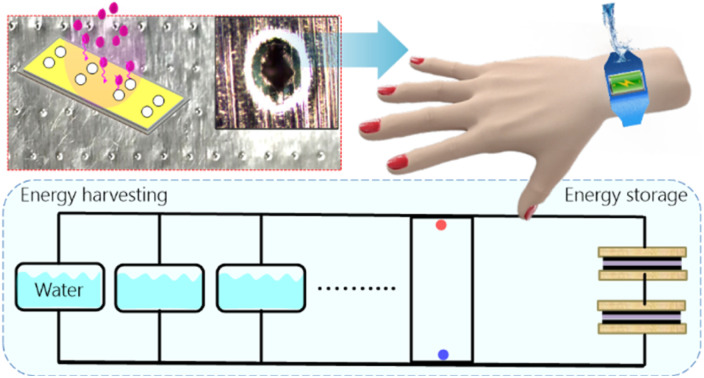

## Introduction

As energy consuming portable and wireless sensors have been rapidly growing, energy harvesting technology harnessing ambient resources has been highlighted as an alternative to produce the electrical power.^1,2^ So far, numerous and intensive efforts have been dedicated to develop wearable and portable energy harvesting devices using diverse environmental resources such as sunlight,^3,4^ electromagnetic radiation,^5^ heat,^6,7^ human motion,^8,9^ and human sweat.^10,11^ Different mechanisms have been explored using photoelectric effect, piezoelectric effect, triboelectric effect, Seebeck effect, electrochemical induction,^12^ and capacitance change.^13^ Typically, these are insufficient, due to instant output power generation and output fluctuation by weather or human body condition.

Meanwhile, power generation from ambient water appears promising due to wide distribution and easy availability in our living environment.^14,15^ To date, most recent research has focused on a moisture-driven electricity production scheme by employing a variety of materials such as protein nanowires,^16,17^ carbon blacks,^18,19^ graphene,^20,21^ graphene oxide,^22^ and titanium dioxide nanowires.^23–25^ In a recent work, to operate low-power temperature and humidity sensors, a moisture enabled wearable form such as a human-breathing medical mask has been demonstrated using conductive carbon black nanoparticles.^26^ Moreover, for practical application, the sewing technique was adopted to produce a wearable fiber-based moisture energy harvester using conductive conjugated polymer.^27^ Furthermore, based on natural evaporation induced electricity, a report demonstrated continuous output power generation for 100 h when a device with exposed active layer to air was placed in a container that was filled with water in ambient conditions.^28^ However, it is hard to recharge the commercial energy storage devices using the low output current of about 150 nA, induced by the narrow electrode area for proton transfer. Considering that human activities are ranging from static to dynamic motions in variable humidity condition, the approach using water droplets for electricity generation is relatively less affected by external or body conditions.^29^ Also, the device design that is consistently exposed to air, cannot be easily packed for mobile and portable electronic devices. Following these early demonstrations, a variety of hydrovoltaic systems have been designed to improve electricity generation efficiency through material and structural innovations. Cao *et al.*^30^ proposed nanocellulose/carbon-nanotube aerogel-based generators that effectively sustained evaporation-induced charge separation by utilizing ambient-dried porous frameworks, achieving continuous and stable operation. Similarly, a sulfonated cellulose nanocrystal/CNT hybrid device^31^ introduced strong ionic conductivity and mechanical flexibility through a chemically cross-linked polymer network, which enabled long-term and deformation-tolerant output. Yoon *et al.*^32^ further developed a transpiration-driven electrokinetic platform based on PEDOT:PSS/DVS-coated yarns, emphasizing integration into textile structures for wearable energy harvesting. In another approach, Lai *et al.*^33^ realized a multifunctional hydrovoltaic device capable of both electricity generation and fire sensing by employing a photoactive PPy composite. Although these studies have significantly advanced evaporation- and moisture-driven hydrovoltaic technologies, most of them still depend on continuous water evaporation or humidity gradients, and their absolute current outputs generally remain below 100 µA. These constraints limit direct energy storage compatibility and long-term usability in portable or encapsulated systems. Therefore, developing a water-droplet driven energy harvesting device in portable and wearable form remains a challenge for satisfying both reliability and high output power generation in ambient conditions.

In the present work, we introduce a portable platform to drive small electronic devices that consume mW–W power by charging supercapacitors. Specifically, in this work, we used an active polymeric layer, consisting of PVA [poly(vinyl alcohol)] with PSSA [poly(4-styrenesulfonic acid)] as a proton source, and PSSA-MA [poly(styrene sulfonic acid-*co*-maleic acid)] as a cross-linking agent, which form a cross-linked network. In overall area, the acitive layer is sandwiched between a perforated Al electrode and flat Al electrode. Also, we adopted the trichloro(1*H*,1*H*,2*H*,2*H*-perfluorooctyl) silane monolayer to render chemical resistance to the electrodes. The fabricated energy harvesting devices show reliable output generation over 110 h in ambient condition both night and day. In addition, 10 devices were simply combined to enlarge the output performance, generating an output voltage of 1.4 V in serial connection, and high output current of 1.2 mA in parallel connection. Thanks to the reliable and high output generation, the charging voltage of 3.2 V was achieved using four 220 mF supercapacitors. As a result, a 60 mW color-changing light emitting diode (LED) and a multifunctional electronic device that displays clock, temperature, and humidity were operated using the supplied power source from supercapacitors. The approach introduced here provides a simple, low-cost, and scalable route to fabrication of a high output performance water-driven platform for powering portable or wearable sensors and electronic devices.

## Experimental section

### Preparation of energy harvesting device

Conductive Al thin film pieces were prepared by cutting Al roll. Then, fluorinated Al surface was formed after O_2_ plasma treatment and FOTS vapour phase deposition as used for above preparation of hydrophobic Al electrode. The surface of Al was cleaned by oxygen plasma (O_2_: 100 sccm, RF Power: 100 W, *t*: 120 s). During this process, the reactive chain ends with hydroxyl (–OH) group are formed on the surface of the Al surface. In turn, target molecules are covalently bonded with the hydroxyl end group ensuring the strong adhesion between target molecules and the Al surface. The plasma treated Al thin films are put in a sealed container by dropping a few trichloro(1*H*,1*H*,2*H*,2*H*-perfluorooctyl) silane (FOTS) drops (97%, Sigma-Aldrich) that play a role in offering water repellent hydrophobicity. Then, the sealed container was placed in an oven for 95 °C for vaporization of FOTS drops. During the vaporization, the hydrophilic ITO surface is modified into a hydrophobic surface by self-assembling chemical vapour phase reaction between the hydroxyl groups and FOTS head groups (–Cl). We used the ITO (surface resistivity 60 Ω sq^−1^, electrode quality)-coated PET substrate. The oxygen vacancy donates free electrons to the ITO as the electrode. During the O_2_ plasma treatment process, the amount oxygen species of ITO are increased and more hydroxyl (–OH) groups are formed on the top of PET surface. In turn, these seamlessly bond with the FOTS molecules during the vapor deposition, assuring the chemical stability for long-term operation. Next, for the directional water transport through pores, Al surface was processed by using a punch press, rendering pores of 1 cm in diameter. Next, for proton-releasing material as active layer, a composite solution was stirred using a magnetic stir bar at 1000 rpm for 1 h, which consists of poly(vinyl alcohol) (*M*_w_ 89 000–98,000, Sigma-Aldrich), with poly(4-styrenesulfonic acid) (25 wt% in H_2_O, Sigma-Aldrich) as proton source and poly(styrene sulfonic acid-*co*-maleic acid) (PSSA-MA) (*M*_w_ ∼20 000, Sigma Aldrich) as a cross-linking agent, which form a cross-linked network. The proton-releasing active layer (5 cm × 5 cm) was coated on the hydrophobic porous Al, followed by drying at 110 °C for 30 min. Lastly, hydrophobic Al as the top electrode was attached onto the active layer.

### Characterizations

The morphology of various films was characterized by field emission scanning electron microscopy (SEM, SU5000, Hitachi). The crystal structures of various films and mC were analyzed by a high resolution powder X-ray diffractometer (XRD, SmartLab, Rigaku). The chemical bonding within the composites and PSSA was analyzed by Fourier-Transform infrared spectroscopy (FT-IR, Nicolet iS50, Thermo Fisher Scientific Instrument). The open-circuit output voltage and short-circuit output current were measured by using a high resistance electrometer (6517A, Keithley). The 10 samples were measured for their current and voltage output, and the reliability of the performance was examined.

## Results and discussion

### Operation and output performance

**Fig. 1:**
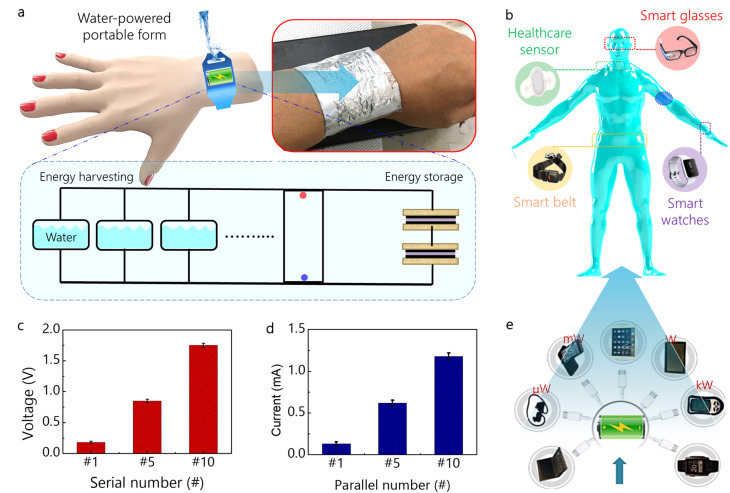
Portable platform for power supply and output performance of water-driven energy device. (a) Representation of portable platform with energy harvesting and storage for driving electrode devices. (b) Power consuming wearable smart electronic devices on human body. Output performance using single, five, and ten device. (c) Measured output voltage in serial connection by varying a number of device using single, five, and ten (d) measured output current in the parallel connection. (e) Required power consumption level from small sensors with µW to high powered microwave oven with kW.

The portable platform for powering small electronics is schematically shown in Fig. 1a and includes the water-driven energy harvesting devices (WEHDs) and energy storage devices. WEHDs scavenge water droplet resource and effectively convert it into direct current (DC) output. Thus, the DC electricity is compatible with storage devices such as supercapacitors or batteries for practical use. Also it is more straightforward in comparison to triboelectric nanogenerators harvesting dynamic human motion, which generates alternating output (AC) output. With conventional TENGs, a power management circuit is necessary for converting the AC signal into DC form to charge and supply it to electronic devices for multifunctional purposes (Fig. 1b). As indicated in Fig. 1c and d, the output performance of WEHDs was investigated by measuring open-circuit voltage (*V*_oc_) and short-circuit current (*I*_sc_). Also, the scalability of output performance was investigated by connecting a number of devices in serial or parallel configuration using three different connections: single, five, and ten devices. As shown in Fig. 1c and d, *V*_oc_ of ∼0.2 V and *I*_sc_ of ∼0.13 mA were observed from single WEHD. Obviously, the output performance was proportionally increased up to 1.8 V from 10 devices in the serial connection and 1.2 mA from 10 devices in the parallel connection. The generated output current of a single device was three orders of magnitude higher in comparison to a previously reported one with ∼150 nA.^28^ Especially, device structure consisting of Al thin film electrode and polymeric active layer is shape-conformal on wrist skin as wearable form for powering portable electronics on human body. The body-attachable DC power source can be compatible with energy storage devices using supercapacitors. Using this DC-form of electrical energy, as shown in Fig. 1b and e, functional devices that use µW–W power level can be operated including sensors, displays, data processors, and wireless transmitters for personal, portable, and wearable applications.

### Design and preparation of framework

**Fig. 2:**
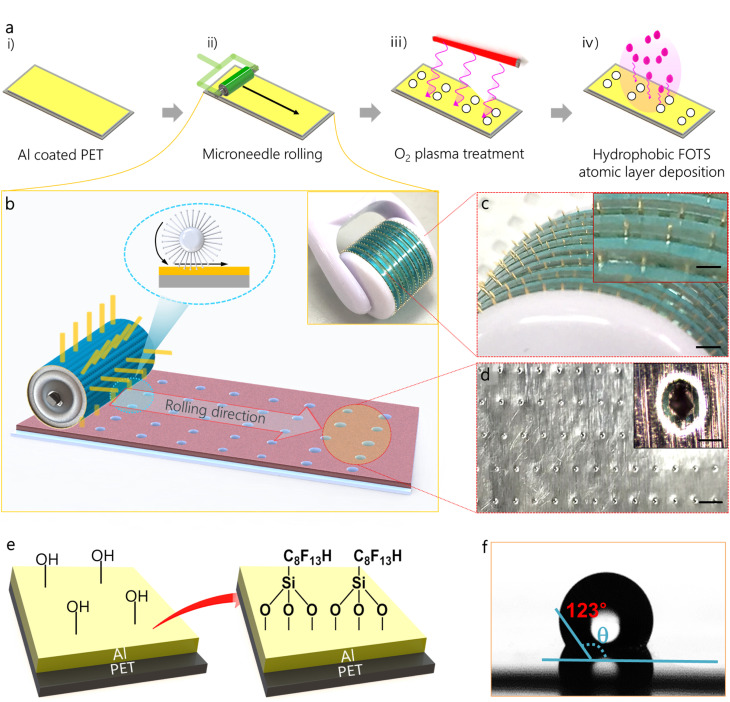
Design and preparation of the electrode. (a) Fabrication process of hydrophobic surface-treated Al coated PET substrate for water transport through pore. (b) Microneedle rolling process for uniform pore formation on Al surface. (c) A used microneedle with 200 µm in diameter. (d) Optical image, showing the micron-sized pore on Al surface. (e) Surface functionalization process using oxygen plasma treatment and self-assembly vapore deposition. (f) Measured contact angle using FOTS coated Al surface, showing hydrophobic property.

**Fig. 3:**
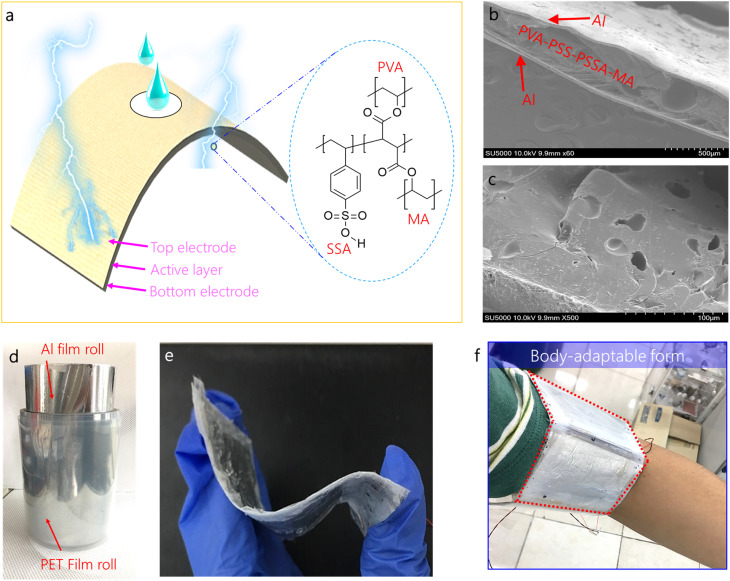
(a) Schematic illustration of device structure using conducting polymeric active layer. (b) A magnified view, showing the sandwiched structure, composed of a perforated Al electrode, PVA/PSSA-MA composite, and flat Al. (c) Porous morphology of integrated polymeric active layer. (d) Al and PET film in the form of roll. (e) Twisted device, showing the flexibility. (f) Body-adaptable form as an wearable electronics.

Device fabrication process was presented in Fig. 2a. The electrode of WEHDs was first designed to transport water molecules as the input energy source through surface pores. Briefly, to this end, a conductive Al thin film (50 µm in thickness) was prepared and subsequently, as shown in Fig. 2b, the uniform hole pattern was readily formed on the Al surface by using a micro needle-type press roller (Fig. 2a(ii) and c). The uniform hole pattern is shown in Fig. 2d and its magnified view of the hole (50 µm × 200 µm) and the line pattern (1 mm × 2 mm) is shown in Fig. S1. Then, O_2_ plasma treatment was performed to render hydrophilic radical (−OH) on the Al surface (Fig. 2a(iii)). Lastly, the prepared Al surface was functionalized using trichloro(1*H*,1*H*,2*H*,2*H*-perfluorooctyl) silane (F_13_-TCS silane, FOTS) by vapour deposition^34^ (Fig. 2a(iv)). Though flexible and low-cost Al electrode in the form of a spool is good choice, which is suitable for large-scale fabrication, degradation can occur, caused by oxidation or corrosion for long-term interaction with water. Here, owing to the stably bonded fluorocarbon bonds (CF_2_ and CF_3_) with strong chemical resistance for bases and acids, FOTS coated Al electrodes are effectively prevented from oxidation or corrosion during water interaction for electricity production; thereby, enabling long-term operation. As shown in Fig. 2e, the hydroxyl (−OH) groups on the Al surface were placed in a sealed container with a few drops of trichloro(1*H*,1*H*,2*H*,2*H*-perfluorooctyl) silane and then the container was kept in an oven for 95 °C for 1 h. During this treatment, covalent bonding by chemical vapor phase reaction is formed between the hydroxyl groups and FOTS head groups (−Cl). In order to verify the functionalized treatment, the surface was analyzed by using water contact angles (*θ*) by the static sessile drop method (Fig. 2f). Obviously, the hydrophobic property (*θ* = 123° > 90°) was observed in FOTS-functionalized PET (F-PET) surface, due to the contribution of nonpolar (–CF_3_) end groups in FOTS with low surface energy.^35^ In contrast, a smaller contact angle (*θ* = 82°) was measured using the pure Al sample without hydrophobic treatment (Fig. S2). Next, as shown in Fig. 3a, a polymeric active layer of 250 µm is sandwiched between the top and the bottom hydrophobic-treated Al electrodes. Fig. 3b and c show the morphology of the integrated device structure, composed of poly(vinyl alcohol) with poly(4-styrenesulfonic acid) as proton source and poly(styrene sulfonic acid-*co*-maleic acid) as a cross-linking agent, which form a cross-linked network. The amorphous nature of the PVA/PSSA-MA composite was comfirmed by using X-ray diffraction (XRD) analysis, showing broad peaks at 2*θ* = 22° (002).^36^ (Fig. S3). In addition, Fourier-transform infrared (FT-IR) spectroscopy was employed to further confirms organic bond states of PVA/PSSA-MA composite. The broad peak that is present at 3436 cm^−1^, showing water absorbed in PSSA.^37^ Other peaks were located at 672, 774, 1006, 1035, and 2927 cm^−1^, are ascribed to C–H out of plane bending, C–H wagging, aromatic ring, SO symmetrical stretching, and C–H stretching. Details of fabrication were described in the experimental method. This fabrication approach is simple, low-cost, and suitable for large-scale production that can employ a roll-to-roll process (Fig. 3d). As shown in Fig. 3e and f, the fabricated WEHD devices are bendable and twistable. The layer of thin Al/polymer/thin Al with thickness of 380 µm is roughly curved and it allows shape-conformable assembly on body skin – *e.g.* wrapping easily around the human wrist for wearable applications.

### Working principle of water-driven electricity generation

**Fig. 4:**
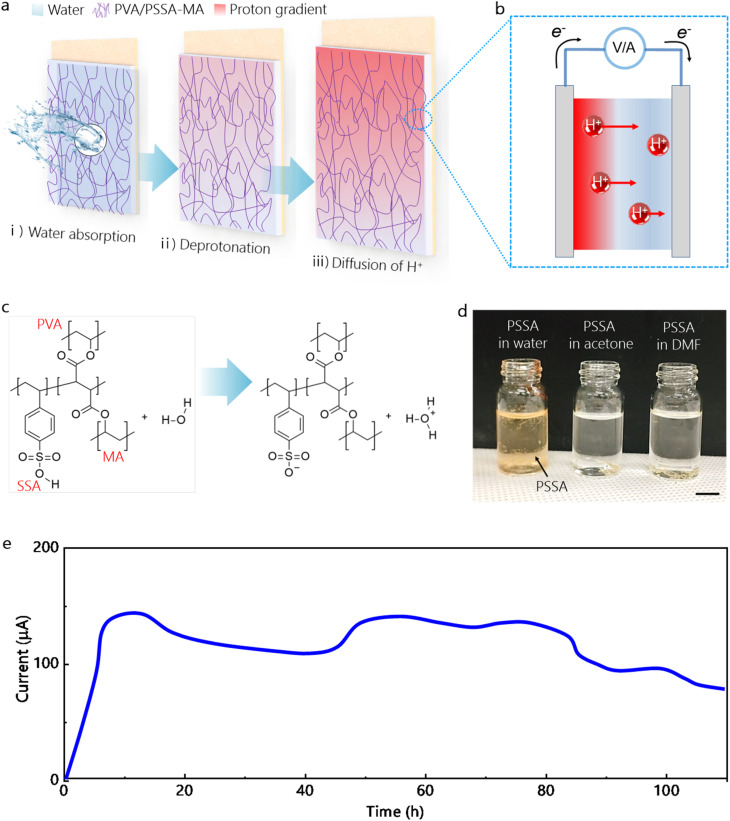
(a) Schematic representation of electricity generation mechanism using conducting PSSA-MA. (b) Output current generation by electron movement. (c) Deprotonation process by reaction with water molecules. (d) Visual photograph, verifying the water-driven proton release. (e) Measured output current for 110 h.

Fig. 4 presents the overall power generation mechanism. In this approach, a rational material combination is indispensable to produce high output power and ensure physical and chemical reliability. Here, we adopted a proton-conducting cross-linked polymeric network that is capable of generating water-driven electricity. Specifically, it consists of poly(vinyl alcohol) (PVA) as water absorber with sulfosuccinic acid (SSA) as proton supplier^30,38^ and styrene sulfonic acid poly(styrene sulfonic acid-*co*-maleic acid) (PSSA-MA) as cross-linking agent.^31,39^ As shown in Fig. 4a, the sequential step of generating electricity includes water absorption, proton generation, and proton diffusion. Water molecules are initially absorbed by passing through the micron-sized pores on the Al surface of the device (Step i, Fig. 4a) and protons are formed by a deprotonation process (Step ii, Fig. 4a) where the SSA reacts with water molecule: SO_3_H + H_2_O → SO_3_^−^ + H_3_O^+^. Then, mobile protons diffuse from the proton rich area at the top to the ion-free area at the bottom, induced by an ion concentration gradient (Step iii, Fig. 4a). The SSA is an actual proton supplier, but it itself has weak chemical stability to water, thus PSSA-MA and PVA were composited to form a robust chemical cross-linking network, ensuring long-term output generation. As a result, as shown in Fig. 4b, the potential difference is produced between the top and the bottom electrodes. Also, the electrons move to neutralize the potential difference through an external circuit, generating current flow. In this process, the structure of interpenetrating polymers each other (Fig. 4c) is robust due to chemically crosslinked network^33^ during water absorption (the bottom, Fig. S5) while sulfonated functional groups are highly reactive with water moleculeS for effective proton exchange. In the case of samples without the chemical crosslinking, the active layer is degraded when the water is in contact with the polymeric layer (the top, Fig. S5). As displayed in Fig. 4c and d, to visually verify water-driven proton release, pure PSSA film is added in different solutions using water, acetone, and dimethylformamide. Among these, color change from transparent to bright orange was observed from the water containing sample, which means the protons are created by the interaction between SSA and water. As indicated in Fig. 4e, a DC output of ∼0.15 mA was sustained for 110 h as the whole body of WEHD was simply dipped in a water container (Fig. S6). Above all, the long-term stability is ensured, owing to the assembly of FOTS protective layer to Al electrode. Typically, when the metal electrode such as Al layer is repeatedly in contact with the electrolytic state of composite by water absorption, the Al is converted into Al_2_O_3_ or other oxide forms that lose its conductivity and thereby decreasing the stable output performance. Additionally, prior to the FOTS depositon, we covered the sides of Al electrode using a tape. In this regard, the PVA/PSSA-MA polymeric layer is tightly adhered to the sides of Al electrode. Also, there are nanopores between FOTS molecules that have F-13 arrangement structure in the vertical direction. The polymeric solution could enter through the tiny gap and the polymeric chains are connected each other. In turn, the approach using the conducting polymer composite greatly extends material choice for fabricating high performance water energy harvesting devices. For the assessment of the overall reliability of the data, one, five, and ten samples were compared for their current and voltage output (Fig. S7) and deviation of individual samples under five repetitive cycles is shown for current and voltage output (Fig. S8). When each sample was tested repeatedly, the deviation of output voltage signal is small. Occasionally, the degree of spreadness on output current signals is significant from the mean value. Overall, the deviation of measured current was larger than that of voltage. When a composite polymer interacts with water, the ion diffusion could be affected by several factors such as swelling, ion transport, change of interfacial conductivity. In comparison, the voltage tends to reflect redox potential shifts and these are smaller in changes.

### Scale-up of electricity and storage

**Fig. 5:**
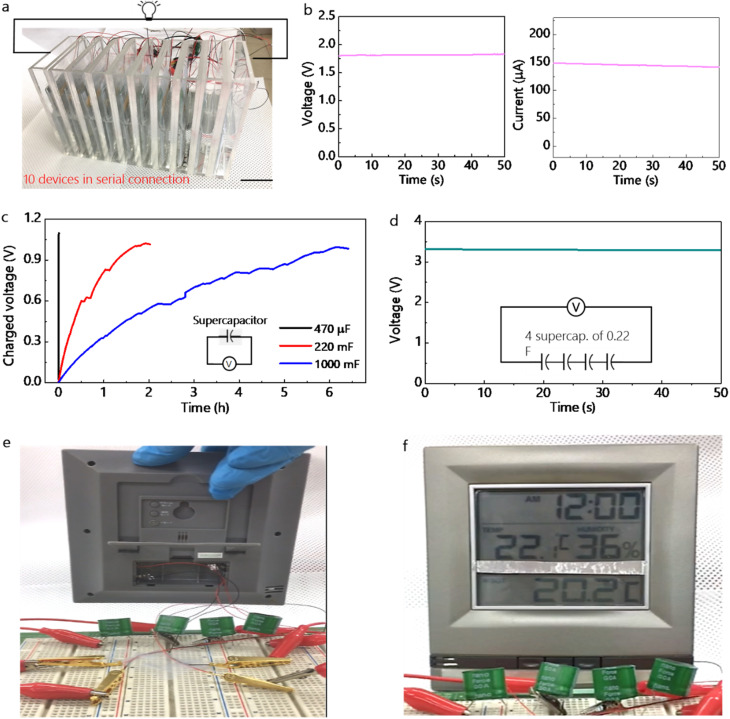
Energy storage and practical applications. (a) Scale-up of electricity using 10 devices in serial connection. (b) Generated output voltage of 1.5 V. and (c) output current of 160 µA. (c) Charging of capacitor of 470 µF or supercapacitor of 200 mF and 1000 mF. (d) Stored electrical energy up to 3.2 V using four supercapacitors. (e) Backside of an electronic display without the external commercial battery. (f) Operation of an electronic display with clock, temperature, and humidity function.

In Fig. 5, to demonstrate the capability of the DC-form reliable and high output power source of WHEDs, we attempted to charge using supercapacitors for practical use. As shown in Fig. 5a, using adopted simple fabrication, many WHEDs can be easily prepared and here the serially connected 10 devices were combined to increase the output voltage up to 1.75 V, generating output current of ∼0.15 mA from the single device (Fig. 5b). Fig. 5c shows electrical charging performance using an energy storage capacitor of 470 µF or supercapacitor of 220 mF and 1000 mF. Output voltage of 1.0 V was quickly achieved in the short time period of 4 s using the 470 µF capacitor and it takes more using supercapacitor with high capacity of 220 mF for 1.45 h to 1.0 V and 1000 mF for 6.5 h to 1.0 V, respectively. Thanks to a reliable charging characteristic, electrical energy was stored up to 3.2 V (Fig. S9) using four supercapacitors of 200 mF in serial connection (Fig. 5d). Thus, as displayed in Fig. 5e and f, stored electrical energy was enough to turn on an electronic device (Fig. 5e, showing no external commercial power source) that displays digital clock, ambient temperature, and humidity sensing (Movie S1). Consequently, these results demonstrate the practical role of developed water energy harvesting devices for operating electronic devices. Importantly, WEHDs introduced here are more suitable for practical application because the device design using water droplet or dipping is not sensitive to external weather conditions, allowing long-term output generation in ambient environments. In a previously reported one,^19^ the generated output is sensitively fluctuated by difference of ambient temperature, wind and humidity.

### Comparative discussion on power generation performance

Recent hydrovoltaic and water-driven energy harvesters show diverse mechanisms and output metrics depending on structural design and interfacial ion transport pathways. For example, the yarn-type PEDOT:PSS/DVS-based TEPG reported by Yoon *et al.* achieved a maximum volumetric power density of 112 µW cm^−3^ with an output current of ∼10 µA per yarn, mainly driven by water transpiration along silk microchannels.^32^ Lai *et al.* introduced a Triton-X/PPy-coated cotton system that produced an open-circuit voltage of 0.42 V and a short-circuit current of 20 µA, corresponding to a power density of 56.6 µW g^−1^, though its current fluctuated with ambient humidity and light exposure.^33^ A sulfonated CNC/CNT hybrid HVEG showed a *V*_oc_ of 0.55 V and *I*_sc_ of 60 µA with 73.5 µW cm^−3^ volumetric power density under evaporative flow conditions,^31^ whereas the nanocellulose/CNT aerogel device by Cao *et al.* generated 697 mV and 10 µA (0.57 µW cm^−2^) under steady-state evaporation.^30^

In comparison, the present WEHD provides a higher absolute current output and more practical energy delivery rather than high localized current density. A single WEHD generates a *V*_oc_ of ∼0.2 V and *I*_sc_ of ∼0.13 mA (130 µA), which is 2–10 times greater than most evaporation- or transpiration-driven systems operated under similar ambient conditions. More importantly, the array of ten devices yields 1.8 V and 1.2 mA, directly charging large-capacity supercapacitors (220 mF–1000 mF) up to 3.2 V without additional rectification or DC–DC conversion. This continuous DC output at the mA-level distinguishes the present WEHD from previously reported nanowire- or aerogel-based generators that typically operate in the µA range and show strong output fluctuations.

Furthermore, the WEHD maintains its performance for over 110 h of continuous operation owing to the FOTS-protected aluminum electrode and cross-linked polymeric proton conductor, while previous devices often exhibit degradation within 24–48 h due to swelling, oxidation, or structural collapse. Therefore, the present WEHD demonstrates a unique combination of long-term DC stability and high total energy throughput, achieving practical charge storage and power supply performance that surpasses most hydrovoltaic systems reported to date.

## Conclusions

We have designed a perforated Al electrode type of wearable energy harvesting device, consisting of PVA [poly(vinyl alcohol)] with PSSA [poly(4-styrenesulfonic acid)] as proton source and PSSA-MA [poly(styrene sulfonic acid-*co*-maleic acid)] as cross-linking agent. Due to a chemical cross-linking network and FOTS surface functionalization, the designed device shows continuous output power generation over 110 h. Charging was viable for supercapacitors with maximum capacitance of 1000 mF, showing its feasibility for charging supercapacitors with different capacitances. In turn, the stored electrical energy using supercapacitor of 200 mF has been demonstrated to drive 60 mW electronic display with clock, temperature, and humidity function. This approach introduced here provides a simple, reliable, and scalable route to fabricate high-performance water energy harvesters. Lastly, future work is aimed at further improving the reliability of the performance, particularly in the current output which shows significant deviations on occasional instances.

## Author contributions

Vinod V. T. Padil: conceptualization, methodology, validation, supervision, writing – review & editing/Sung-Ho Shin: conceptualization, methodology, visualization, writing – original draft, writing – review & editing/Min Wook Pin: formal analysis, methodology, investigation/Joon Ha Chang: formal analysis, investigation/Hyeon Do Shin: formal analysis/Daniel M. Mulvihill: validation, writing – review & editing/Jiamu Qian: writing – review & editing/Paolo Matteini: methodology, validation/Byungil Hwang: validation, investigation, writing – review & editing/Tae Gwang Yun: investigation, validation, formal analysis, writing – review & editing/Jun Young Cheong: conceptualization, methodology, supervision, resources, writing – original draft, writing – review & editing/Il-Doo Kim: resources, data curation, supervision, writing – review & editing, funding acquisition.

## Conflicts of interest

The authors declare no competing interests.

## Supplementary Material

RA-015-D5RA04176G-s001

RA-015-D5RA04176G-s002

## Data Availability

The data supporting this article have been included as part of the supplementary information (SI). Supplementary information is available. See DOI: https://doi.org/10.1039/d5ra04176g.
