# Supplementary material for: Water droplet-driven and perforated conducting polymer composite energy harvester: platform for powering portable and wearable electronics
Source: RSC Adv. 2025 Dec 15;15(58):49689–98. doi: 10.1039/d5ra04176g (PMC12703487; doi:10.1039/d5ra04176g)
Supplement: RA-015-D5RA04176G-s001 [file RA-015-D5RA04176G-s001.pdf]

Supplementary Information (SI) for RSC Advances.
This journal is © The Royal Society of Chemistry 2025

Supporting information for:

Water droplet-driven and perforated conducting polymer composite energy harvester: Platform for powering portable and wearable electronics

Vinod V. T. Padil[1,+], Sung-Ho Shin[2,+], Min Wook Pin[3], Joon Ha Chang[4], Hyeon Do Shin[5], Daniel M. Mulvihill[6], Jiamu Qian[6], Paolo Matteini[7], Byungil Hwang[8,*], Tae Gwang Yun[9,*], Jun Young Cheong[6,*], Il-Doo Kim[2,10*]

[1]School of Nanoscience (SNS), Central University of Gurajat (CUG), Kundhela-391107, Vadodara, Gujarat, India

[2]Department of Business Incubation Center, Korea Advanced Institute of Science and Technology, 193 Munji-ro, Yuseong-gu, Daejeon 34051, Republic of Korea.

[3]Analysis and Assessment Research Group, Research Institute of Industrial Science and Technology (RIST), 37673, Republic of Korea

[4]Department of Materials Science and Engineering, Korea National University of Transportation, Chungju 27469 Republic of Korea

[5]Department of Material Science and Engineering, Polymer Division, Graduate School of Chungnam National University, 99 Daehak-ro, Yuseong District, Daejeon, Republic of Korea

[6]James Watt School of Engineering, University of Glasgow, Glasgow, G12 8QQ, UK

[7]Institute of Applied Physics "Nello Carrara", National Research Council, 50019 Sesto Fiorentino, Italy

[8]School of Integrative Engineering, Chung-Ang University, Seoul 06974, Republic of Korea

[9] Department of Molecular Science and Technology, Ajou University, Suwon 16499, Republic of Korea

[10] Department of Materials Science and Engineering, KAIST, Daejeon 34141, Republic of Korea

[+]: These authors contributed equally to this work.

Corresponding author: bihwang@cau.ac.kr (Prof. B. Hwang) / ytk0402@ajou.ac.kr (Prof. T. G. Yun) / JunYoung.Cheong@glasgow.ac.uk (Dr. Jun Young Cheong) / idkim@kaist.ac.kr (Prof. Il-Doo Kim)

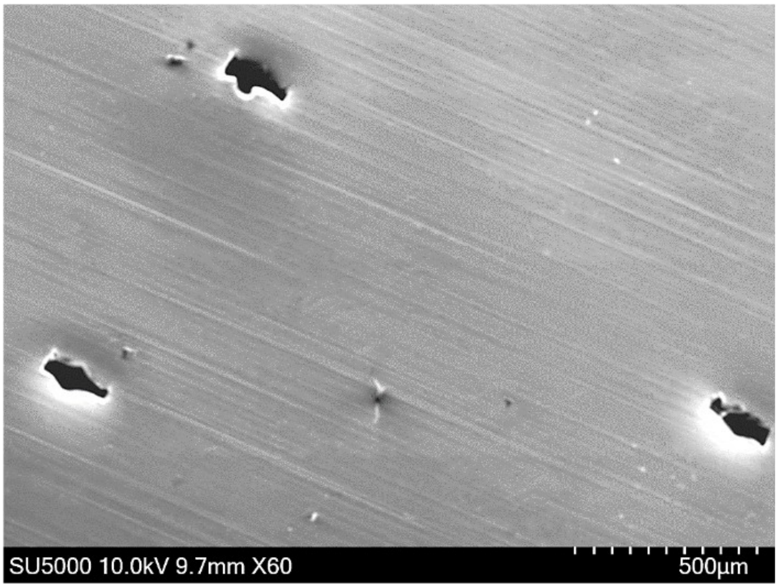

Fig. S1. SEM image, showing the prepared Al electrode by using microneedle roller.

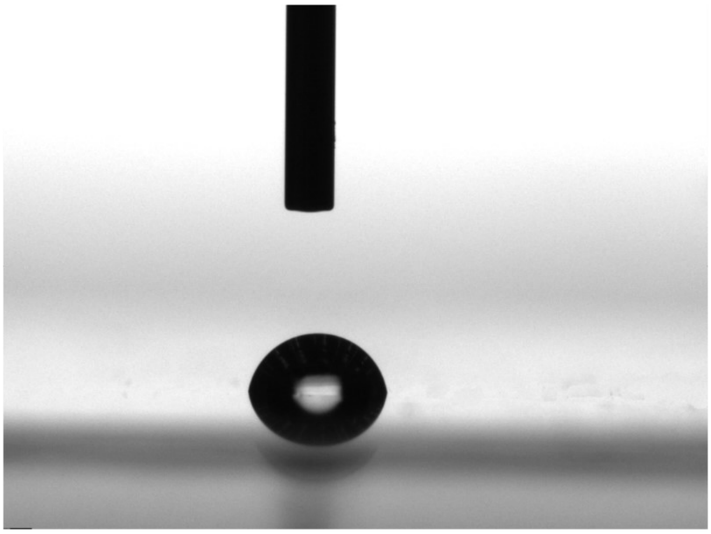

Fig. S2. Water contact angle measurement, showing the hydrophilic property of sample without the functionalization FOTS treatment.

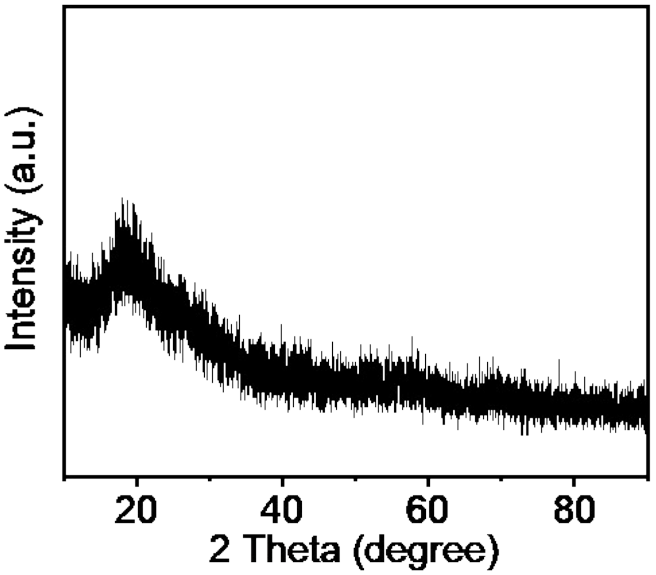

Fig. S3. XRD patterns of PVA/PSSA-MA composite showing its amorphous nature.

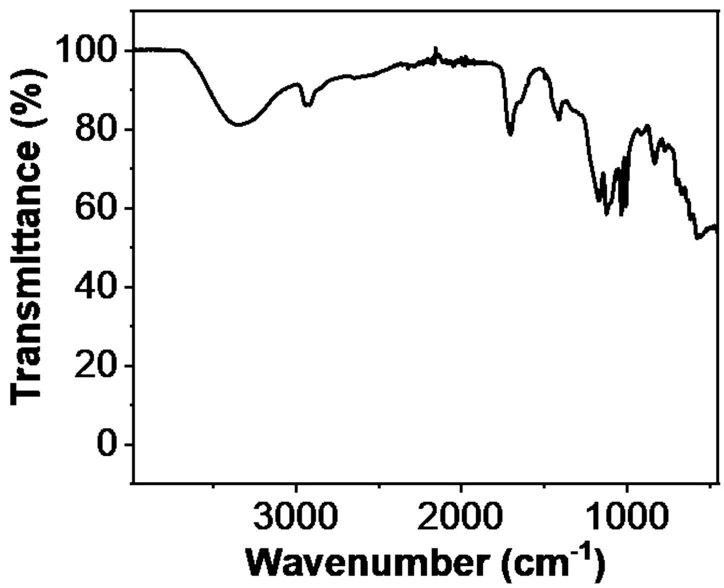

Fig. S4. FTIR curve of PVA/PSSA-MA composite.

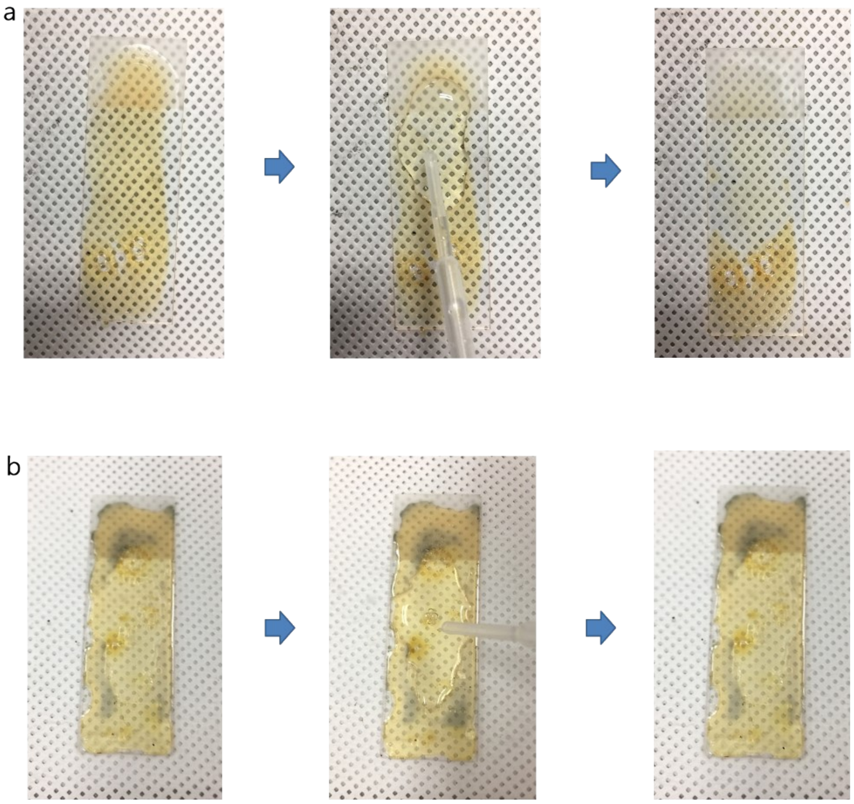

Fig. S5. Photograph of the different composite. (a) Poor durability of sample based on PSSA only. (b) Durable sample by water contact, attributed to the crosslinked network.

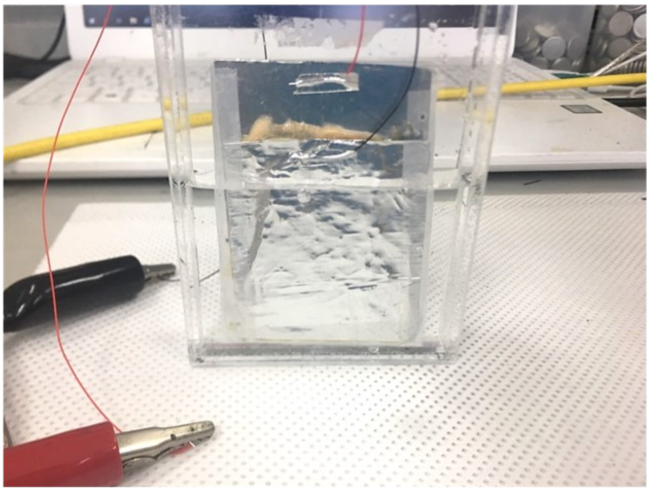

Fig. S6. (a) Photograph, showing the output measurement in dipping state.

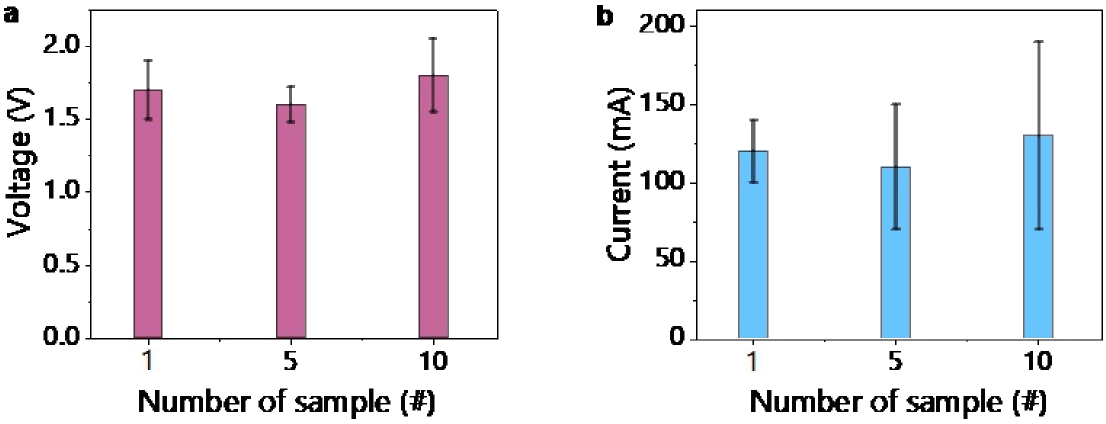

Fig. S7. Measurement data, showing the deviation using 1, 5, 10 samples. (a) Output voltage deviation. (b) Output current deviation.

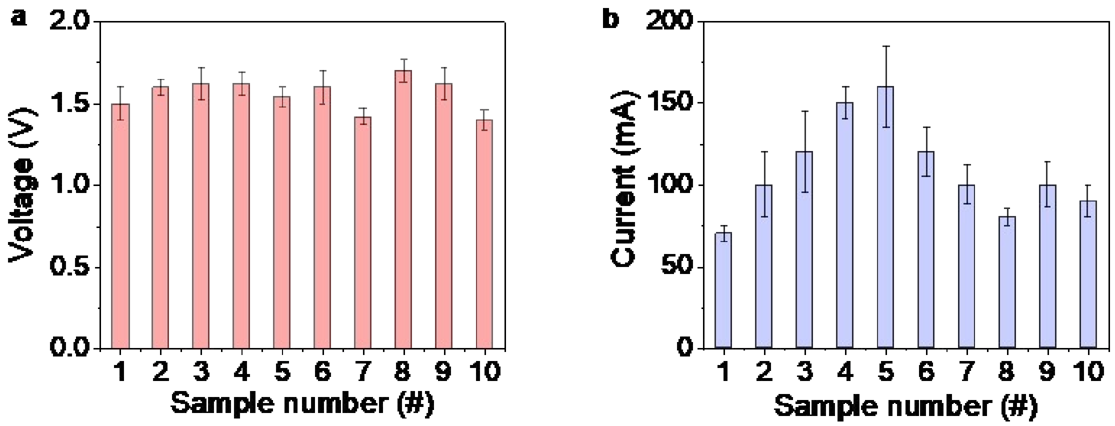

Fig. S8. Measurement data, showing the deviation under five repetitive cycles for each samples. (a) Output voltage deviation. (b) Output current deviation.

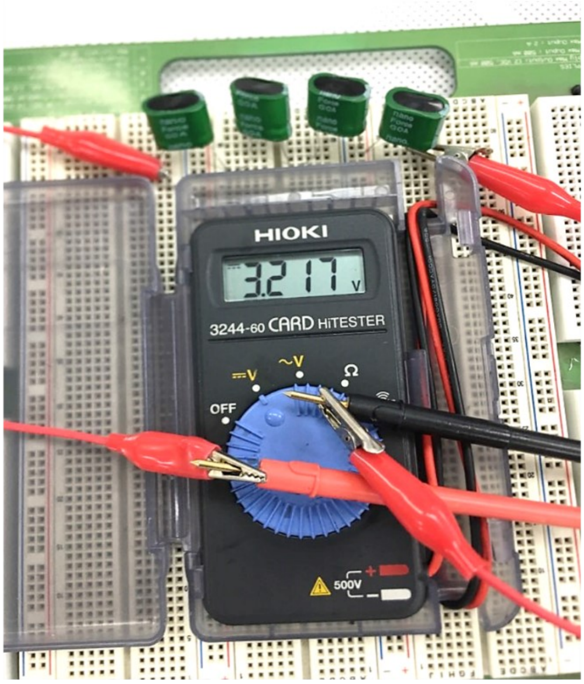

Fig. S9. Charged output voltage using four supercapacitors of 200 mF in serial connection.
